# Insanity in Switzerland

**Published:** 1848-01-01

**Authors:** 


ART. IX.—*Ueber das öffentliche Irrenwesen in der Schweiz (Mit einem Antrag an die Hauptversammlung der Schweiz gemeinnützigen Gefellschaft vom September,* 1846, *eingereicht in Namen der Direktions-kommission der St. Gallisch-Appenzell. Section derselben von deren Vorstand.)* J. M. HUNGERBÜHLER, Regierungsrath. St. Gallen und Bern. 1846.

*On the State of the Insane and of Lunatic Asylums in Switzerland,* &c. By J. M. HUNGERBÜHLER. St. Gall and Bern. 1846.

AT the meeting of Swiss naturalists in 1830, the deplorable condition of the unfortunate individuals afflicted with cretinismus was fully discussed, and since that time Dr. Troxler, in his classical work on "Cretinismus, as *endemic* in Switzerland," has thrown fresh light upon the peculiarities attending this disease. The institution founded by Dr. Guggenbühl for the treatment of cretinismus in Switzerland, has likewise, in a scientific-medical point of view, been of practical importance. In classifying the diseases of the mind *psychologically* and *pathologically,* it will be readily admitted that cretinismus, occupying as it does a very low position among these diseases, has in Switzerland attracted to itself a considerable degree of attention, while the other affections of this class have, in many of the cantons at least, been almost totally overlooked and neglected. It has been asserted, that the state of mental cultivation arrived at among any people may be safely deduced from a consideration of the attention bestowed upon the treatment of mental disorders. Should this assertion hold good, and we are inclined to a certain extent to support it, this state of mental cultivation, in some of the so-called free cantons, must be very low indeed; and in the course of this paper we shall avail ourselves of the opportunity of pointing out the difference of system employed in these different cantons in the treatment of the insane, from which a comparison may be drawn of the actual condition of this, in many respects, important European republic. Deficient as is still the treatment of the unfortunate lunatic in many cantons, the time is, however, fortunately long ago passed by, when, as a criminal, he was exposed to punishment, or as bewitched, or in communication with the Evil One, he was sentenced to torture and death; when, instead of being an object of compassion and pity, he was looked upon with horror and dismay. In the year 1484, Pope Innocent VIII. empowered by the Bull "Summis desiderantes affectibus," the author of the celebrated work, "Malleus maleficarum," to act as Plenipotentiary, Inquisitor, and Judge for the extinction of witchcraft, heresy, and lunacy; in accordance with which commission, within a very few years in the bishoprick of Trier alone, more than 6000 unfortunates were tortured and burnt. This curious work, now become very rare, ought to be considered as a satire upon the understanding of man. Printed first in Cologne in 1489, it likewise contains the "Tenor bullæ Apostolicæ cum approbatione Doctorum almæ Universitatis Coloniensis." As a portion of the papal bull just mentioned, we cannot refrain from quoting a portion indicative of the gross ignorance of the age. We read thus—"Sane nuper ad nostrum non sine ingenti molestia pervenit auditum, quod in nonnullis partibus Alemaniæ superioris, nec non in Maguntinensibus, Coloniensibus, Treverensibus, Salzburgen, sibus et Bremensibus provinciis, civitatibus, terris, locis et diocecibus complures utriusque sexus personæ propriæ salutis immemores et a fide Catholica deviantes cum *dæmonibus incubis et succubis abuti,* ac suis incantationibus, carminibus et conjurationibus aliisque nefandis superstitiis, et sortilegiis excessibus criminalibus et delictis, *mulierum partus, animalium fœtus,* terræ fruges, vinearum uvas et arborum fructus, mulieres, pecora, pecudes et alia diversorum generum animalia, vineas quoque, pomeria, prata, pascua, blada, frumenta, et alia terræ legumina *perire, suffocare et extingui facere* et *procurare,* ipsosque homines, mulieres, jumenta, pecora pecudes et animalia diris tam intrinsecis quam extrinsecis doloribus et turmentis afficere et excruciare ac eosdem homines ne gignere et mulieres ne viris actus conjugales reddere valeant impedire." From the days of Innocent VIII. to Pius IX., what a contrast! From the bigotry, superstition, and cruelty of the former, to the mild and noble virtues of the latter, what a change! From the horrid treatment of the unfortunate insane, legalized in the times of the former, to the gentle kindness, so characteristic of the latter, in the treatment of this miserable portion of our fellow-men in the benevolent institutions of the papal states! How great an alteration from the *dungeons* for the insane in Zurich, in 1721, to the excellent hospital for those persons existing in the same town at present! It is the progress of the age, of the human mind, of Christianity, and may it go on ever progressing! Corresponding to the change in the times are the publications of the different authors of these eras; and the writings of the learned jurisconsults of that day—of Bollwer, Lampert, Torreblanca,

Glanville, Beaumont, and Carpzov, differ greatly from the late productions of Feuerbach, Mittermaier, Wächter, Heffter, Bentham, and Rossi. Great as has been the progress made, there is, nevertheless, much reason to suppose that the science of criminal legislation, connected as it undoubtedly is with mental disturbances, is still in its infancy.

Before proceeding to describe the present state of the insane in Switzerland, let us take a rapid glance over that in several other civilized countries, in order that a comparison may be the more easily drawn. Our readers are too well acquainted with the general condition of the insane in this country to render it necessary for us to dwell more than a moment upon the state of our own lunatic asylums. The former neglect and barbarity of treatment have been superseded by thorough reforms, and Christian as well as scientific care. Suffice it here to observe, that in 1844, to a population upwards of 16,000,000 in England and Wales, the number of insane was more than 20,000, two-thirds of whom were maintained at the expense of the public. In Scotland there were seven, and in Ireland eleven county asylums, supported at great cost. The establishments of St. Luke, Bethlehem, Hanwell, Wakefield, and Lincoln, the asylums at York, Gloucester, Lancaster, Northampton, and Nottingham, and the excellent private institutions in the country, are too well known to be here more than referred to.

Passing from Great Britain to France, we see, that even among the horrors of the great revolution, (verifying in so high a degree the saying of Schiller, that "the most fearful of fearful things is man in his rage,") —even in those terrible days, a ray of pity and of hope penetrated into the prisons of the insane, and led on by that noble man, Pinel, a new land of promise was held forth to the despairing view of the insane. Esquirol followed in the footsteps of his great master; and at length we see that all asylums, whether public or private, have been placed under the inspection of the government. In 1841, the number of insane in France was not far from 20,000, three fourths of whom were in different public and private asylums. It is curious to observe that Napoleon's "code penal" places the insane under the same head as mad dogs and other animals: "Ceux qui laisseront divaguer des *insensés* ou furieux, ou animaux malfaisans ou féroces; ceux qui auront excité ou n'auront pas retenu leurs chiens, lorsqu'ils attaquent ou poursuivent les passans, quand même il n'en serait résulté aucun mal, ni dommage, seront punis, &c." On the other hand, the laws for the protection of the insane, passed in the year 1838, will ever remain as a noble proof to posterity of the attention bestowed by the French administration upon this important subject. The great hospitals of the Saltpetrière and Bicêtre are of European celebrity, and in quitting a hurried survey over the state of insanity in France, we remark with sincere pleasure the formation of the philanthropic "*Societé de Patronage*" in Paris, with the useful co-operation of the "*dames patronesses.*" In the seven provinces of Belgium, we find thirty-seven asylums, all under the inspection of the government. In Holland, in the year 1840, in thirty-seven asylums the number of insane was but 826. In Austria, in 1837, there were but thirty-eight asylums, with 4696 insane patients; and, with the exception of those of Halle and of Prague, the greater part of the Austrian asylums were in a miserable condition. Among the States of the German Confederation, we find Baden with its asylum of Illenau, under Dr. Roller; Wirtemberg with the two establishments of Zwiefalten and Vinnenthal, the last under the care of Dr. Zeller; in Saxony, the long known institutions of Sonnenstein, near Pirna, and of Coldiz. In Bavaria, are the asylums of Baireuth, Bamberg, Würzburg, Frankenthal, Giessing, and the new one at Erlangen. In Nassau, that of Schuberg, and in Saxe Weimar, that of Jena; in Mecklenburg-Schwerin, that at Sachsenberg; and in Hanover, that of Hildesheim. In Hesgen may be mentioned the establishments at Hayna and Merrhausen; in Brunswick, that of the same name; in Frankfort, the ancient asylum of the city, capable of great reform; and in Hamburg, the intended foundation of a large asylum for 400 insane, has only been for the moment laid aside by the great conflagration of that city, a few years back. In Prussia, the so necessary reforms commenced by Langerman and Reil have been ably followed up by Damerow. In the Rhenish provinces of Prussia may be enumerated the institution of St. Thomas, at Andernach, and those of Düsseldorf, Trier, Aachen, and Cologne. Three large asylums are about being completed in the provinces of Saxony, East and West Prussia; in the Brandenburg province, besides the old asylums of Neu-Ruppin and Soraw, a new institution for the insane from Berlin and Potsdam is to be built. A new establishment for the insane has likewise been founded at Halle, and contains about 400 patients. In Silesia, honourable mention is made of the asylums at Leubus, and at Plagwiz and Brieg. In the Great Duchy of Posen is found the asylums of Owinsk; and in Westphalia, that of Marsberg. The Danish asylum at Bistrup is capable of receiving great alteration for the better; and in Norway and Sweden, their active and intelligent ruler, Oscar, has already commenced the necessary changes in the legislation for the insane, as well as that for criminals. In Russia, the present Emperor Nicholas has ordered several institutions to be built: those at St. Petersburg, and at Helsingfors, in Finland, are best known. In Italy, the progress of psychology has corresponded with that of the most advanced countries; and in Upper Italy, we call attention to the institutions of Turin and Reggio; and in Lower Italy, to that of Aversa, near Naples, and of Palermo. In Portugal, Spain, Greece, and Turkey, the state of the unfortunate insane is much the same as it was centuries ago. In Portugal, a certain number of lunatics are admitted into the hospital of St. Jose; and in Constantinople we find *one* asylum for Mahometans, and several for Greeks, Armenians, and Franks. In Egypt, the insane are imprisoned in a portion of the hospital Mohristan, at Grand Cairo. From the dark and dreary scenes of some of these old and decaying countries, let us turn towards the United States of America, where the philanthropist meets with fresh encouragement in the institutions erected for the insane, as well as for the purposes of criminal legislation. While in the latter part of the eighteenth century there existed but *three* ill-supported and miserably maintained asylums for the insane in the States, there are now found upward of twenty new institutions in excellent order, and others are in a state of preparation. South America and Mexico may be considered to stand in much the same relation to the United

States as the institutions of Spain to those of England. A portion of the hospital in the city of Mexico is set aside for the reception of the insane, and in the Brazils there exists one establishment in Rio Janeiro.

Having now cursorily glanced over the different institutions for the insane in various countries, let us return to Switzerland, where we find our author adopting the words of Professor Schröder, at Utrecht, when speaking of the deplorable condition of the insane in Holland:—" Si vero jam ad patriam oculos advertimus, nescimus utrum nos gravius mærore commoveri an pudore suffundi sentiamus." In order to arrive at a clear idea of the state of psychology in the different cantons, our author divides them into *five series.* In the first or *lowest,* where these unfortunate beings are entirely disregarded by the government and police, he places the cantons of *Lucerne, Freiburg, Uri, Schwytz, Unterwalden, Zug, Glarus, Appenzell, Schaffhausen, Ticino,* and *Wallis,*—eleven cantons, with about a third of the population of the whole republic, or above 600,000 inhabitants.

With regard to *Lucerne,* the first and most important of these cantons, we see that throughout their whole legislative code mention is made of the insane but in one paragraph of the police regulations, which says, as late as 1836, that " in cases of lunacy, such people are to be watched and provided for by their friends, or the persons with whom they live, under penalty of a fine varying from *two to sixteen francs.*" In the second canton of importance in this series, *Freiburg,* the great council have come to the determination to build an hospital, attached to which is to be an asylum specially intended for the treatment of those suffering under mental diseases. In Schwytz and Glarus, no movement in favour of reform in this respect has taken place. In Unterwalden, the insane are sometimes, by the desire of their family, shut up in the prisons or houses of correction. In a few of these cantons, those afflicted with mental disease among the poorer classes are sent to the poorhouses or unions, and among the richer classes they are generally sent to the asylums of the neighbouring countries.

In the second series, may be placed the cantons of Solothurn, Baselland, and Graubünden, with a population of nearly 200,000, or about one-eleventh of the inhabitants of the whole country. Solothurn possesses one cantonal establishment, and another in St. Catharina for the city. These two asylums are, upon the whole, tolerably well conducted, but are much too limited for the population, which amounts to 60,000 souls, with an average of from sixty to seventy lunatics. Dr. Ziegler-Oberli has distinguished himself by his endeavours to better the condition of these establishments. Baselland has an asylum for the insane connected with the poorhouse. In Graubünden, the worst classes of lunatics are sent to the house of correction at Fürstenau; but through the generosity of Herr von Tschappina, who has by will left a considerable sum of money towards the erection of a proper asylum, it is hoped that the condition of the mad in this canton will soon be materially improved.

*In the third series,* in which we find the insane no longer placed in poorhouses or prisons, but in hospitals, may be reckoned the cantons of *Zurich, Bern, Aargau, Thurgau,* and *Vaadt,* with a population of almost 1,100,000, or about one-half of that of Switzerland.

That portion of the hospital at Zurich allotted to the insane contains about twenty considered as capable of being restored, and 100 incurables. There are, besides, several private asylums under the care of Drs. Herzer, Fehr, and Schmied.

The Bern hospital contained in 1840, of insane patients remaining from 1839, males, 24; females, 24—in the whole, 48. Received in 1840 17 male and female patients; the aggregate 34. Subjected to treatment in 1840 an equal number, making 82. Cured in the time 26; 12 males, 14 females. Five were sent out uncured—2 males, and 3 females; remaining in the establishment 1. Deaths, 3; 1 male and 2 females. Remaining in 1840, 47 patients—25 males and 22 females.

In 1843 there were eighty-two individuals treated in this hospital, eleven of whom were cured, and four died. Dr. Lehman is the physician to the hospital, which is old, and by no means corresponding with the wealth and high position assumed by the canton of Bern. Among the private asylums may be mentioned that of Dr. Tribolst, near the city of Bern. In *Aargau* the old cloister of Königsfelden has been converted into a hospital, with a portion of it devoted to the treatment of mental diseases, and containing forty-four cells for such cases. There were in the year 1844 here treated thirty-seven males and forty females, or in all seventy-seven, of whom three were cured, three considerably improved in health, and seven died—leaving, on the 1st of January, 1845, sixty-four in the hospital. Dr. Siebold has the care of these patients.

The people of *Thurgau* have in like manner availed themselves of an ancient cloister in which to establish a hospital, with a lunatic asylum, under the care of Dr. Merk.

Already, as early as 1810, the canton Vaadt founded a hospital, with a division thereof devoted to the treatment of insane, and it was decreed that "Dans la maison des aliénés sont recus les aliénés des deux sexes dont l'existence dans leurs familles et dans la société devient pénible et dangereuse, ou qui laissent un espoir probable de guérison." The government or council has lately voted a considerable sum of money for the improvement of the condition of the insane in this canton.

In the *fourth* series, distinguished by the *absolute* separation of the institutions for the treatment and cure of recent cases, and the asylums for the reception of those considered as incurable, Mr. Hungerbühler cannot admit any of the Swiss cantons; but in the *fifth* or last series, where the separation may be said to be *relative* between the two classes just mentioned of institutions for the insane, he places the city portion of the canton of Basel—Genf or Geneva, Neuenburg and St. Gallen, containing together 300,000 inhabitants, or about one-seventh of the whole population of the country. The institution for the insane in Basel contains about seventy patients. That in Genf or Geneva, built according to the plan of our Tuke, the founder of the asylum at Wakefield, contains about sixty-six patients. The name of Coindet is well known in connexion with this establishment. According to him—

| Patients in the Asylum of Geneva, 1st January. | | | Number admitted. | | | | Sent out. | | | | Classified as | | Duration of Treatment. Days. | |
|---|---|---|---|---|---|---|---|---|---|---|---|---|---|---|
| | | | Male. | | Female. | | | | | | | | | |
| Year. | Male. | Female. | First time. | Récidive, or Relapses. | First time. | Récidive, or Relapses. | Cured. | Improved. | Unimproved. | Deaths. | Curable. | Incurable. | Male. | Female. |
| 1834 | 27 | 24 | 11 | 4 | 8 | 3 | 13 | 6 | 2 | 6 | 28 | 49 | 10,334 | 8,418 |
| 1835 | 25 | 24 | 11 | 1 | 11 | 2 | 13 | 2 | 3 | 9 | 13 | 12 | 9,045 | 8,996 |
| 1836 | 23 | 25 | 11 | 3 | 15 | 2 | 11 | 4 | .. | 5 | 17 | 14 | 10,119 | 11,099 |
| 1837 | 28 | 32 | 14 | .. | 17 | .. | 7 | 8 | 5 | 12 | 18 | 13 | 9,878 | 10,769 |
| 1838 | 28 | 30 | .. | .. | .. | .. | .. | .. | .. | .. | .. | .. | .. | .. |
| TOTAL...... | | | 47 | 8 | 51 | 7 | 44 | 20 | 10 | 32 | 76 | 88 | 39,376 | 39,282 |
| | | | 113; Former occupants, 51; Total, 164. | | | | 106; Remaining, 58; Total, 164. | | | | Total, 164. | | | |

From this table we see thus, that of 164 patients 44 were cured (or 27 per cent.); 20 sent out improved in condition (or 12 per cent.); and 32 died (or 20 per cent.) Of the 164 patients, 105, or two-thirds, were unmarried. The mean age of the patients upon entrance was 35 to 36 years. Two-thirds were supported at the public expense. Average daily cost of each patient was 92 centimes; and the mean duration of those cured was 107 days.

In the following table follows an analysis of the different mental diseases in the institution of Geneva during four years:—

| NAME OF DISEASE. | 1834. | | 1835. | | 1836. | | 1837. | | TOTAL. | | |
|---|---|---|---|---|---|---|---|---|---|---|---|
| | Male. | Female. | Male. | Female. | Male. | Female. | Male. | Female. | Male. | Female. | Both. |
| Mania . . . . | 19 | 13 | 13 | 15 | 16 | 19 | 16 | 23 | 64 | 70 | 134 |
| Monomania . . | 8 | 9 | 5 | 10 | 4 | 11 | 9 | 12 | 26 | 42 | 68 |
| Dementia . . . | 6 | 9 | 11 | 10 | 9 | 10 | 9 | 10 | 35 | 39 | 74 |
| Idiotismus . . | 5 | 2 | 7 | 1 | 7 | 1 | 8 | 1 | 27 | 5 | 32 |
| Epilepsia, with complications | 4 | 2 | 3 | 2 | 4 | 4 | 4 | 3 | 15 | 11 | 26 |
| Total . . . | 42 | 35 | 39 | 38 | 40 | 45 | 46 | 49 | 167 | 167 | 334 |

The institution for the insane at Vernet contained on the first of January, 1845, 31 male and 51 female patients, or together, 82. During the year, 45 fresh cases were entered and 37 sent out. There were 12 cured, 7 improved, 7 unimproved, and 9 deaths. At the end of December,

1845, of 90 patients, 78 were considered as incurable, and 12 were hopeful cases. The number of females was much greater than of males. These institutions are under the care of government.

At St. Pirminsberg, the canton of St. Galle possesses an excellent and well conducted institution for 108 insane, those admitted being chiefly cases of mania. In the following table we furnish a concentrated view over the statistics of mental disease in this canton:—

*Tabular View over Mental Diseases in St. Galle, in the year* 1838.

| | Dementia-Idiotismus. | Mania. | Melan-cholia. | Epilepsia and Complica-tions. | Total of Mental Disease. | Average to population of 1000 souls. |
|---|---|---|---|---|---|---|
| Male . . . | 76 | 38 | 15 | 28 | | |
| Female . . | 84 | 46 | 33 | 20 | | |
| Not known . | 137 | 10 | 17 | 22 | | |
| TOTAL . | 297 | 94 | 65 | 70 | 526 | 3·311 |

Of the 297 cases of dementia and idiotismus 143 were paupers, and 154 non-paupers; of the 94 cases of mania 52 were paupers, and 42 non-paupers; of the 65 cases of melancholia 18 were paupers, and 47 non-paupers; and of the 70 cases of epilepsia, &c., 29 were paupers, and 41 non-paupers. In improving the condition of the insane in this canton Dr. Rheiner has been among the foremost.

In now taking leave of Mr. Hungerbühler's work, we cannot refrain from observing the difference in the treatment of the insane existing in the two divisions of the cantons. While in the so-called liberal or radical cantons much attention has, time after time, been bestowed upon the state of these miserable beings, the treatment of them by the primitive or conservative cantons has continued *primitive* indeed. Late important events have directed the eyes of Europe towards Switzerland, and it is earnestly to be desired that the primitive cantons, brought into more immediate contact with the others, may copy the progress made by them in the study of psychology. To remain in their present unenlightened condition would be at once a blot upon the name of a people claiming to rank among civilized nations, and a disgrace to the age in which we live.

